# Editorial: Recent advances on omics and biomarkers research in renal transplantation

**DOI:** 10.3389/fmed.2023.1210249

**Published:** 2023-06-13

**Authors:** Xiaoming Ding, Xiaopeng Hu, Zheng Jenny Zhang

**Affiliations:** ^1^Department of Renal Transplantation, Hospital of Nephrology, The First Affiliated Hospital of Xi'an Jiaotong University, Xi'an, Shaanxi, China; ^2^Department of Urology, Beijing Chao-Yang Hospital, Capital Medical University, Beijing, China; ^3^Department of Surgery, Comprehensive Transplant Center, Feinberg School of Medicine, Northwestern University, Chicago, IL, United States

**Keywords:** renal transplantation, rejection, infection, omics, biomarker

Kidney Transplantation is the preferred treatment for most patients with end-stage renal disease (ESRD) (1). Despite advances in post-transplant immunotherapies, recipients often face acute or chronic rejection, which is a major cause of graft loss (2). Subclinical rejection (SCR), which affects 10–30% of KTx recipients within the first year after KTx, is recognized as early predictors of late graft failure (3). Effective treatment guided by early detection of SCR may preserve renal architect and improve long-term graft survival. In addition, acute kidney injury (AKI) following transplant is associated with increased risk factor for AR. However, it is challenging to identify early changes before irreversible injury to the graft occurs. While biopsy of transplanted kidney tissue remains the gold standard for detection of SCR and clinical rejection, it is an invasive test that has several limitations, including (1) sampling error, (2) subjective nature of biopsy slide reading, (3) high costs, and (4) risk of major post-biopsy complications. Therefore, there is a need for developing novel non-invasive diagnostic tools and monitoring strategy. The overarching goal for the collection, Recent Advances on Omics and Biomarkers Research in Renal Transplantation, is to showcase recent studies on discovery of more effective biomarkers for early detection and intervation of graft rejection. We thank all authors for their valuable contributions to the topic, and the reviewers for their insightful comments. The proceedings on the topic highlight new approaches to biomarker discovery that have the potential to lead to more effective treatment strategies for future clinical research.

Biomarkers are biomolecules found in body fluids or tissues. Traditional monitoring after renal transplantation includes the continuous measurement of serum creatinine (Scr), estimated glomerular filtration rate (eGFR), blood concentration of immunosuppressive drugs, donor specific antibody (DSA) monitoring, virus quantity detection, and so on. However, they often reflect advanced tissue damage and have limited ability to accurately detect SCR. Recent advances in high-throughput molecular biotechnologies and bioinformatics have now made it possible to in-depth analysis of kidney allograft rejection and response to treatment. Accordingly, new biomarkers can be identified and validated by non-invasive or minimal-invasive approaches. And the discovery of novel biomarkers as reported in this collection raised new hope for early detection and development of promising new treatment approaches.

Most transplanted kidneys come from deceased donors and inevitably experience ischemia reperfusion injury, which may lead to AKI and affect the recovery of renal function after surgery. Three machine learning algorithms used the study by Li et al. include LASSO, random forest, and support vector machines. Six hub genes (MDFI, EHBP1L1, FBXW4, MDM4, RALYL, and ESM1) were identified as potential predictors of acute kidney injury. These genes can be used as a new tool for early diagnosis of AKI.

Transplant recipients must rely upon the lifelong use of immunosuppressive agents to protect transplanted organ from rejection. Insufficient immunosuppression may increase the risk of allograft rejection in kidney transplant recipients. The occurrence of antibody-mediated rejection (ABMR) and T cell-mediated rejection (TCMR) can significantly lead to deterioration of graft function and eventually graft loss. Currently, complement activation in the form of C4d deposits in peritubular capillaries (PTC) is used as an ABMR marker (4). However, not all ABMR cases are positive for C4d. Raïch-Regué et al. used immunohistochemistry to show that p-S6RP in the peritubular capillary endothelia was associated with circulating human leukocyte antigens (HLA)-DSA in transplanted kidney tissue biopsies and first suggested that it could be used as a sensitive alternative biomarker for HLA-DSA to diagnose ABMR when DSA was not available or not yet detected. Renal operational tolerance, defined as the condition in which a SOT recipient retains stable graft function and lacks histological signs of rejection, after at least 1 year of immunosuppressant discontinuation (5), is a rare but beneficial condition. To investigate the underlying mechanism of this phenomenon, Massart et al. identified the variation of HOMER36, IQCH, and LCN36 in two tolerant patients and three controls by using exome sequencing technology. The variation of these genes occurred in the primary villus structure, suggesting that this may be related to renal immune tolerance. Johnson et al. reviewed recent advances in systems biology techniques and focused on the development of transplant immunology as a result of these techniques. Their analysis of the literature shows great potential for initial applications of multiomics and broad research transplants of emerging biomarkers. For example, based on the development of sequencing technology, single nucleotide polymorphisms (SNPs) can be used to distinguish the origin of cfDNA, so that donor derived cell free DNA (dd-cfDNA) can be widely used to detect the early graft function status (6). They also suggest that although the application of systemic immunology to transplantation may provide new insights into disease diagnosis and treatment, there are still some limitations.

Viral infections have been considered to be an important factor responsible for the deterioration of graft function. BKV is a common posttransplant opportunistic viral infection among transplant recipients (7). Over the last decade, studies on the possible biomarkers of EVs (microvesicles/exosomes) in urine have received some attentions (8). Bruschi et al. performed proteomic analysis of urine EVs samples from 29 patients with BKV infection and 15 controls with normal renal graft function. It was found that DNASE2, F12, AGT, CTSH, C4A, C7, FABP4, and BPNT1 could achieve the maximum differentiation between the KTR of BKV and controls, indicating that these proteins may be used as early diagnostic indicators after BKV infection.

The development of new omics technology has also brought great progress to the traditional methods of kidney transplantation detection. One of the advantages of single-cell RNA sequencing (scRNA-seq) is that it can identify different immune cells in the graft kidney, which is of great significance to explore the changes both in number and function of macrophages (9), T cells, and B cells (10) during rejection, and could be helpful to find new therapeutic targets. Urine is regarded as the most accessible and non-invasive source of specimens. At present, a large number of omics techniques have been applied to study mRNA, non-coding RNAs (ncRNAs) and proteins in urine in order to find new detection markers and therapeutic targets. In addition, people have gradually realized the importance of non-HLA antibodies in graft rejection (11). Currently, Angiotensin type 1 receptor (AT1R), Major Histocompatibility Complex-Class I–related chain A (MICA) antibodies are mainly studied. However, the lack of standardized testing has greatly limited the wide application of non-HLA antibodies. The development of omics technology can provide a new means of detection, which is conducive to the subsequent development of targeted therapies to treat these rejection reactions, thus improving the survival rate of grafts.

In conclusion, identification of earlier diagnostic biomarkers will not only allows designing individualized therapy for timely therapeutic intervention, but also further advance understanding of pathgeneisis of kidney allograft rejection. The field has been evolving rapidly; substantial prograss has been made over the last decade. It is conceivable that as the number of clinical studies using omics and other innovative approaches continues to increase, biomarkers with high sensitivity and specificity will be identified and vlidatated in the next few years, thus guiding the development of early preventive treatment strategies for clinical applications.

## Author contributions

XD wrote and revised the editorial. XH and ZZ revised the editorial. All authors reviewed the manuscripts submitted to the Research Topic and the final version of this editorial. All authors contributed to the article and approved the submitted version.
